# Local deformation and stiffness distribution in fly wings

**DOI:** 10.1242/bio.038299

**Published:** 2019-01-15

**Authors:** Henja-Niniane Wehmann, Lars Heepe, Stanislav N. Gorb, Thomas Engels, Fritz-Olaf Lehmann

**Affiliations:** 1 Department of Animal Physiology, Institute of Biological Sciences, University of Rostock, Albert-Einstein-Str. 3, Rostock 18059, Germany; 2 Department of Functional Morphology and Biomechanics, Zoological Institute, University of Kiel, Christian-Albrechts-Platz, 24118 Kiel, Germany; 3 Laboratoire de métérologie dynamique, Department Géosciences, Ecole Normale Supérieure and PSL, Paris, 24 rue Lhomond, 75231 Paris Cedex 05, France

**Keywords:** Insect, Wing mechanics, Wing stiffness, Flight, Stiffness scaling, *Drosophila*, *Musca*, *Calliphora*

## Abstract

Mechanical properties of insect wings are essential for insect flight aerodynamics. During wing flapping, wings may undergo tremendous deformations, depending on the wings’ spatial stiffness distribution. We here show an experimental evaluation of wing stiffness in three species of flies using a micro-force probe and an imaging method for wing surface reconstruction. Vertical deflection in response to point loads at 11 characteristic points on the wing surface reveals that average spring stiffness of bending lines between wing hinge and point loads varies ∼77-fold in small fruit flies and up to ∼28-fold in large blowflies. The latter result suggests that local wing deformation depends to a considerable degree on how inertial and aerodynamic forces are distributed on the wing surface during wing flapping. Stiffness increases with an increasing body mass, amounting to ∼0.6 Nm^−1^ in fruit flies, ∼0.7 Nm^−1^ in house flies and ∼2.6 Nm^−1^ in blowflies for bending lines, running from the wing base to areas near the center of aerodynamic pressure. Wings of house flies have a ∼1.4-fold anisotropy in mean stiffness for ventral versus dorsal loading, while anisotropy is absent in fruit flies and blowflies. We present two numerical methods for calculation of local surface deformation based on surface symmetry and wing curvature. These data demonstrate spatial deformation patterns under load and highlight how veins subdivide wings into functional areas. Our results on wings of living animals differ from previous experiments on detached, desiccated wings and help to construct more realistic mechanical models for testing the aerodynamic consequences of specific wing deformations.

## Abstract (precis)

**Summary:** We determined mechanical properties of wings under load in three fly species. Fly wings broadly deform similar to homogenous beams. Veins and joints shape wing mechanics and govern wing deformation during flapping flight.

## INTRODUCTION

Most insect wings are flexible, non-cambered, flat structures, producing aerodynamic forces for locomotion during gliding and wing flapping at elevated frequencies. They consist of thin membranes and ambient, longitudinal and cross veins (Brodsky, 1994; Chapman, 1998). The wing membrane is composed of multiple layers of cuticle that interconnect wing veins (Gorb et al., 2000; Song et al., 2007; Ma et al., 2017). Veins greatly vary in size and shape between animal species and determine the wing's structure and mechanical behavior under load (Wootton, 1981; Combes and Daniel, 2003a; Appel et al., 2015). Veins may also carry nerves from innervated setae and campaniform sensilla (Gnatzy et al., 1987), situate accessory hearts to supply body appendages with hemolymph (Pass, 2000), and primarily contribute to wing mass.

Flexibility of insect wings prevents mechanical damage and is required in various behaviors, such as grooming and flight. In flight, wings are deformed by inertial-elastic, aerodynamic and viscous forces. Inertial-elastic forces, for example, are prominent at stroke reversals and viscous damping helps to prevent the flexible trailing edge from fluttering (Combes and Daniel, 2003c). Since wing shape determines the wing's aerodynamic performance, any deformation of the surface during flapping motion may change flow conditions and thus lift and drag production (Young et al., 2009; Zheng et al., 2013). Previous findings on the significance of wing flexing for flight are, however, inconsistent. Compared to rigid wings, flexible wings may change the direction of forces (Zhao et al., 2010), maximize total lift production (Moses et al., 2017) and enhance force production into the downward direction (Mountcastle and Daniel, 2009; Zhao et al., 2010; Nakata and Liu, 2012; Mountcastle and Combes, 2013). By contrast, flexible models of hoverfly wings produce less lift than stiff model wings (Tanaka et al., 2011). In forward flight, flexible wings augment the lift-to-drag ratio mostly owing to wing twist and not changes in wing camber (Zheng et al., 2013). Besides passive deformation, wings of insects such as dragonflies, locusts and flies are thought to be supplemented by a series of muscles that allow some measure of active control of wing deformation (Ellington, 1984).

Computational models of fruit fly wings with reinforced leading edges, moreover, suggest higher lift-to-drag and lift-to-power ratios than wings with uniform stiffness distribution or rigid wings (Nguyen et al., 2016). A recent two-way fluid-structure interactions (FSI) model on bumblebee flight, by contrast, implies that model wings with uniform stiffness produce more lift and thrust than wings with a stiffness distribution similar to a genuine bumblebee wing. This is due to the hyper-compliant wing tip that stabilizes flight but at the cost of elevated aerodynamic power requirements (Tobing et al., 2017). The latter findings were confirmed by an experimental study on bumblebees with artificially stiffened wings that lead to more flight instabilities during forward flight compared to controls (Mistick et al., 2016). Further evidence for the above findings is provided by numerical results on aerodynamic power requirements for flight with flexible wings (Nakata and Liu, 2012). Explanations for the above contradictions have recently been discussed elsewhere (Fu et al., 2018).

The wing's vein network predominately determines wing bending and twisting behavior. In flies, for example, the v-shaped profile of the leading wing edge resists bending but may easily twist when applying force behind the torsion axis (Ennos, 1988). This twist may propagate to the rest of the wing, resulting in an overall change of camber. Cambering increases with decreasing branching angle between the v-shaped veins, while immobilization of the wing base prevents camber formation under load. Under the latter conditions, torsion is greatly reduced (Ennos, 1988). Change in corrugation is thus thought to be a typical result from bending-torsion control in insect wings (Sunada et al., 1998; Rajabi et al., 2016a).

Wing bending and flexing at vein joints depend on several factors including the shape of veins, the existence of vein spikes and also on the distribution of resilin (Weis-Fogh, 1960). The latter findings have recently been demonstrated in numerical models on vein joint mechanics (Rajabi et al., 2015) and in a study on various types of resilin-mediated wing joint mechanics in the dragonfly *Epiophlebia* (Appel and Gorb, 2011). Resilin is not only present in wing vein joints but also in the internal cuticle layers of veins (Appel et al., 2015). Besides the number and thickness of cuticle layers, material composition and cross-sectional shape, resilin predominately determines vein material properties and thus the degree of elastic deformation. By contrast, flexible membranes between veins may increase structural stiffness under load and thus the integrity of insect wings (Newman and Wooton, 1986). The latter finding was also demonstrated by finite element modeling of corrugated wings during out-of-plane transversal loading (Li et al., 2009). Other mechanical features of insect wings include dorso-ventral anisotropy (Combes and Daniel, 2003a,b; Ma et al., 2017; Ning et al., 2017) and a gradient in wing stiffness from base to tip (Steppan, 2000; Lehmann et al., 2011; Moses et al., 2017). Since spanwise is typically larger than chordwise stiffness (Combes and Daniel, 2003a; Ning et al., 2017), wings often twist at the stroke reversals when forces peak within the flapping cycle (Ning et al., 2017).

In most previous studies, wing stiffness is quantified by Young's modulus *E,* spring constant *k* and flexural stiffness *EI* with *E* the Young's modulus and *I* the wing's second moment of area. While Young's modulus describes the relationship between tensile stress and tensile strain within the material, the spring constant describes the ratio between the deflection and loading force, and flexural stiffness is a measure that combines material and shape properties. On average, Young's modulus of insect wings amounts to 5 GPa (Vincent and Wegst, 2004) but may greatly vary from tens to hundreds of Megapascal in flies and dragonflies (leading wing edge, Chen et al., 2013; Tong et al., 2015) and even in different parts of the wing (Haas et al., 2000a,b; Rajabi et al., 2016b). Spring stiffness covers measurements between ∼1 Nm^−1^ in butterflies (Mengesha et al., 2011) and ∼50 Nm^−1^ for the wing base of blowflies (Ganguli et al., 2010; Lehmann et al., 2011). Typical measures for flexural stiffness range from ∼10^−9^ Nm^2^ at the wing tip of blowflies (Lehmann et al., 2011) to ∼5×10^−3^ Nm^2^ at the wing base of moth (Combes and Daniel, 2003b). Although above parameters are mainly species-specific (Combes and Daniel, 2003a), a large part of the variance is explained by dry-out effects during the measurements. Wing stiffness greatly increases as wings desiccate. This leads, for example, to an approximately sixfold increase in flexural stiffness in butterflies (Steppan, 2000), an ∼10-fold increase in shear stiffness of larval fly cuticle (Vincent and Wegst, 2004), an ∼20-fold increase of Young's modulus in dragonflies (Chen et al., 2013) and an approximately twofold increase in spring stiffness of wings of painted lady butterflies (Mengesha et al., 2011). Altogether, this suggests that measurement conditions are crucial for any reconstruction of complex wing models based on stiffness recordings (Herbert et al., 2000; Combes and Daniel, 2003b).

In this study, we investigate the scaling of mechanical behavior of wings attached to their living bodies in three species of flies, i.e. fruit flies, house flies and blowflies. We quantify the impact of dry-out effects on wing shape, estimate anisotropic deformation and score local deformation of the entire wing surface while loading wings at various sites using a micro-force transducer. From these data, we calculate the wing's flexural stiffness and spring constants along selected bending lines. The measured data eventually allow us to construct an advanced, numerical model of fly flight using computational fluid dynamics and fluid-structure interaction. This model is currently under development and will provide quantitative results on flow patterns, aerodynamic forces and moments during flapping of model fly wings with a stiffness distribution similar to that of the natural archetype.

## RESULTS

### Wing shape and desiccation

**Fig. 1.**
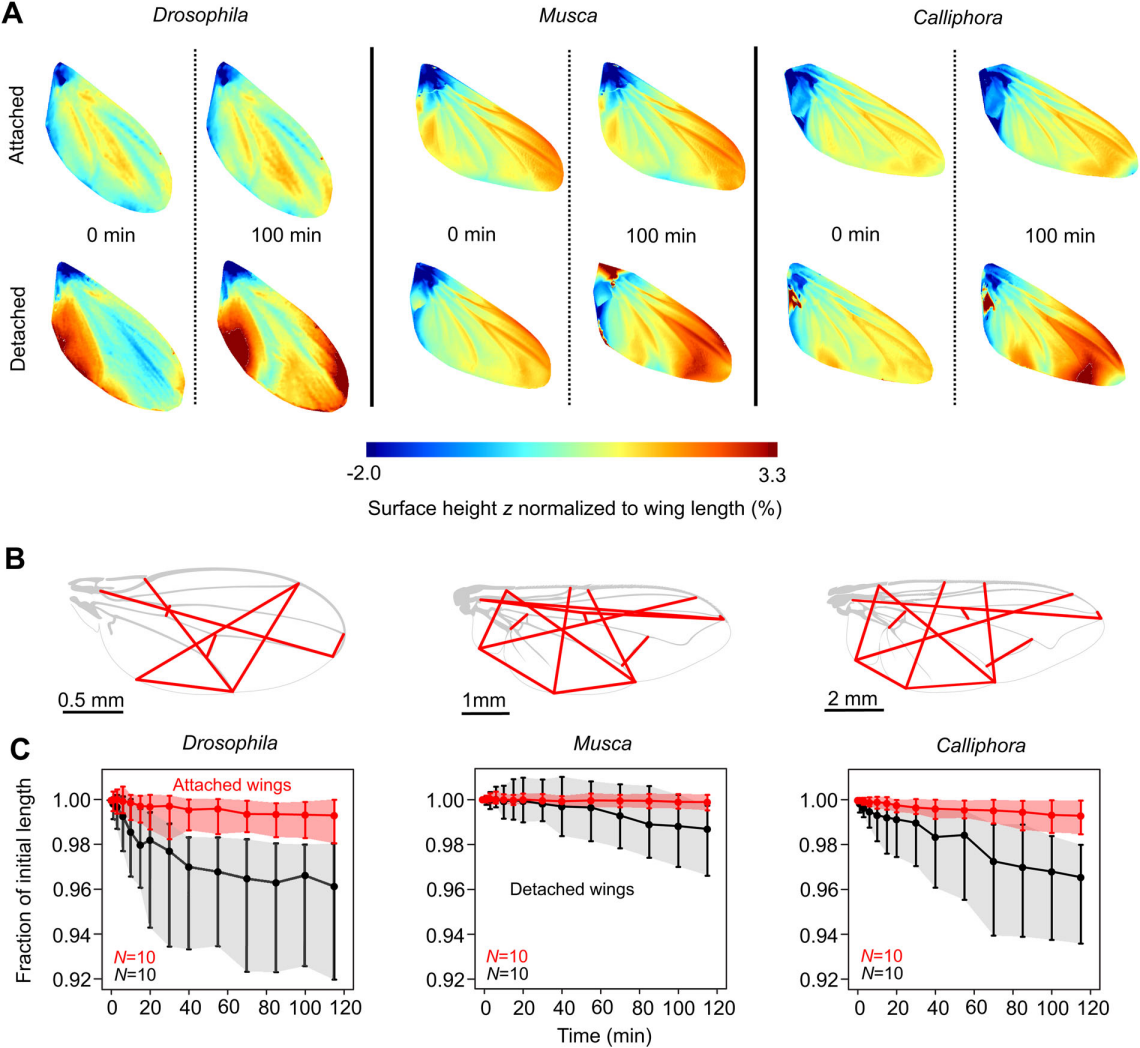
**Wing surface changes during desiccation.** (A) Initial surface height values (*z*-values) and after 100 min in wings attached to intact, living animals (upper row) and detached wings (lower row). Surface values are normalized to wing length i.e. ∼2.15 mm in *Drosophila*, ∼6.28 mm in *Musca* and ∼9.29 mm in *Calliphora* (Table 1). (B) Lines (red) for estimation of wing deformation during desiccation. Wing images are captured by a photo camera from the top. (C) Mean relative length change of lines in *B* in detached wings (black) and wings of intact animals (red). Data are medians and shaded areas indicate lower and upper quartiles. *N*, number of tested flies.

To demonstrate the difference in wing properties in detached wings and wings that are attached to the living animal, we scored the shape changes using profilometer measurements (Fig. 1A) and a geometrical analysis (Fig. 1B,C). Fig. 1A shows the changes in structure of single wings in all three tested fly species immediately after preparation (0 min) and after 100 min (see Table 1 for environmental conditions). Wings that are attached to intact animals deform only little in *z*-direction with local changes of not more than ∼1% wing length within the time period (upper row, Fig. 1A). By contrast, in wings that are detached from the animal body, surface *z*-values distinctly change, reaching up ∼±3.3% wing length (lower row, Fig. 1A). Mean absolute change in *z*-direction was significantly different between attached and detached wings (Welch two sample *t*-test, *P*=0.043, *N*_attached_=15 wings, *N*_detached_=14 wings). Assuming that the observed changes in wing surface are correlated with the hydration condition of the wing and this in turn with wing stiffness, our data suggest that measurements of stiffness in detached wings are less reliable compared to measurements in wings of intact animals.

**Table 1.**
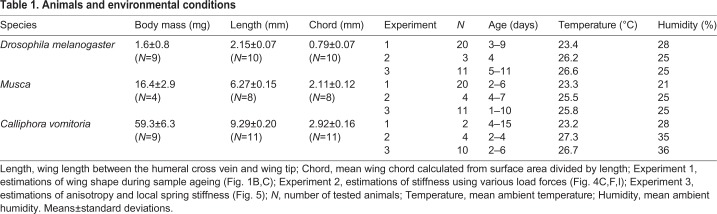
**Animals and environmental conditions**

Fig. 1B,C shows the relative changes in distance between distinct morphological wing points (red lines) projected onto the image plane of a photo camera and for a larger data sample (Table 1). Data show that in all tested animal species with intact/attached wings, sample ageing is negligible. After 100 min measurement time, mean length of the scoring lines changed less than 1% of their initial length (model I, linear regression; slope, −1.28×10^−4^ min^−1^, −0.16×10^−4^ min^−1^, −1.10×10^−4^ min^−1^; *N*, 1112, 1988, 1764 from 10 flies each; *Drosophila, Musca, Calliphora,* respectively). Detached wings, by contrast, show more significant changes in length of scoring lines of up to ∼3% (slope, −5.08×10^−4^ min^−1^, −1.54×10^−4^ min^−1^, −5.29×10^−4^ min^−1^; *N*, 1112, 1890, 1833 from 10 flies each; *Drosophila, Musca, Calliphora,* respectively). On average (0–120 min), the median relative distance between morphological markers (red lines, Fig. 1B) in detached wings is significantly smaller than in attached wings (Wilcoxon signed rank test with continuity correction, *P*<0.001, *N*=39 measurements). Moreover, in all tested species, the interquartile ranges of length change in detached wings are significantly larger than in attached wings (Wilcoxon signed rank test with continuity correction, *P*<0.001, *N*=39 measurements).

### Stiffness dynamics during wing loading

**Fig. 2.**
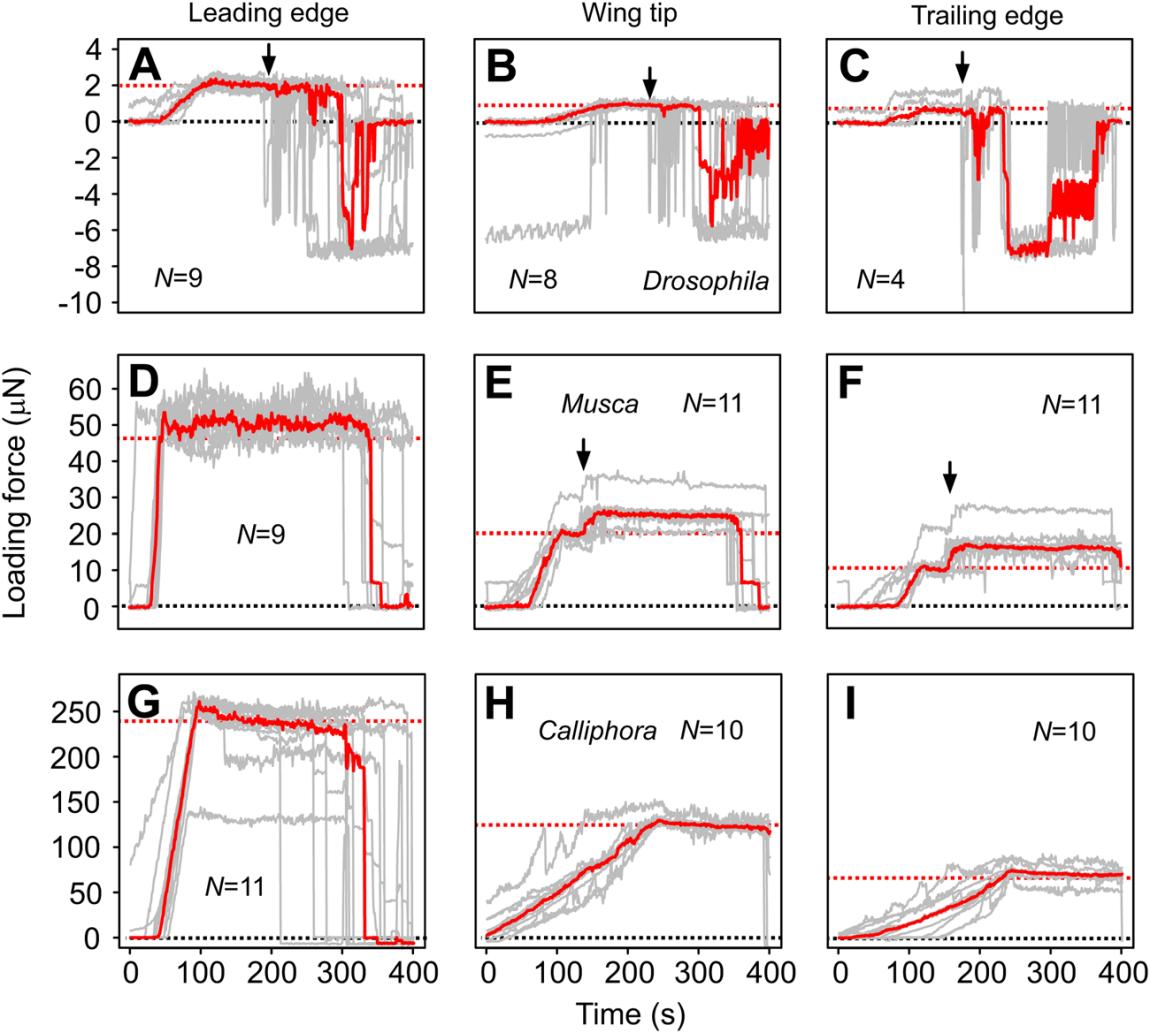
**Dynamics of the wing deformation in response to force loading and unloading.** Force-time curves during dynamic loading experiments in *Drosophila* (A–C), *Musca* (D–F) and *Calliphora* wings (G–I). Wings are loaded at point 1 (wing leading edge; A,D,G), point 3 (wing tip; B,E,H) and point 7 (all species, wing trailing edge; C,F,I). Single runs are shown in gray, medians of all runs in red. Red dashed lines indicate desired load at which sensor movement approximately stops. Arrows indicate onset of visual patterns projected on the wing during profilometer measurements. Under the latter conditions, force may increase presumably owing to some thermal drift of the sensor during the surface scan. *N*, number of tested flies.

**Fig. 3.**
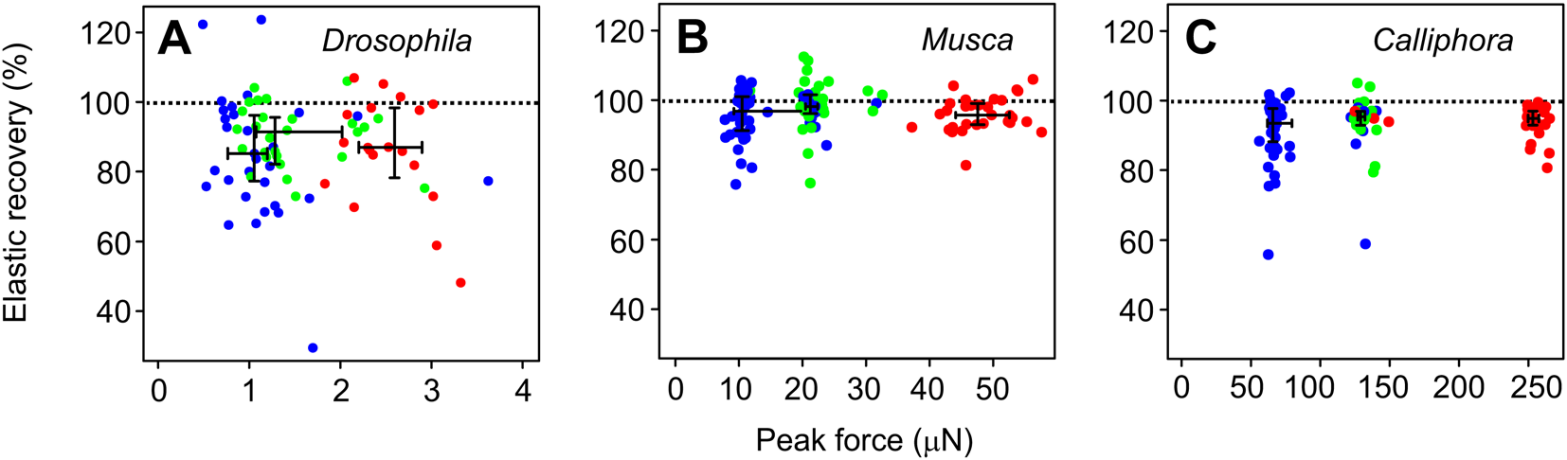
**Recovery of elastic potential energy (elastic recovery) during wing loading.** The measure was calculated from the ratio between maximum forces at stimulus phase I and mean force of phase II (see Materials and Methods) in the three tested species. Colors indicate measurements in three regions of the wing. Red, leading wing edge area (*N*=18 in A, *N*=29 in B, *N*=31 in C); green, wing tip area (*N*=24 in A, *N*=33 in B, *N*=27 in C); blue, trailing wing edge area (*N*=36 in A, *N*=54 in B, *N*=48 in C). *N*, number of tested animals. Medians, lower- and upper quartiles are shown in black.

Fig. 2 shows the recorded force traces at three selected load points in all tested flies. Most of these traces show force peaks during the loading process that slightly decrease and stabilize afterwards. This might indicate that the sensor's wire tip slightly slides on the cuticle. However, the decrease might also result from creeping deformation and thus a loss of elastic potential energy, owing to the visco-elastic properties of resilin. To quantify the force change, we calculated the ratio between mean peak force at phase 1 and mean force at phase 3 (means of five measurement values, Fig. 3). Averaged over the results of all 11 testing sites, elastic recovery (force ratio) was 0.87±0.15 (*N*=78 measurements in 11 animals) in *Drosophila*, 0.93±0.06 (*N*=116, 11 animals) in *Musca* and 0.93±0.08 (*N*=106, 10 animals, means±standard deviation) in *Calliphora*. In all cases, the relative loss of force was not more than ∼13% and significantly different from zero (Welch one-sided *t*-test, *P*<0.001, three species).

### Spring and flexural stiffness

**Fig. 4.**
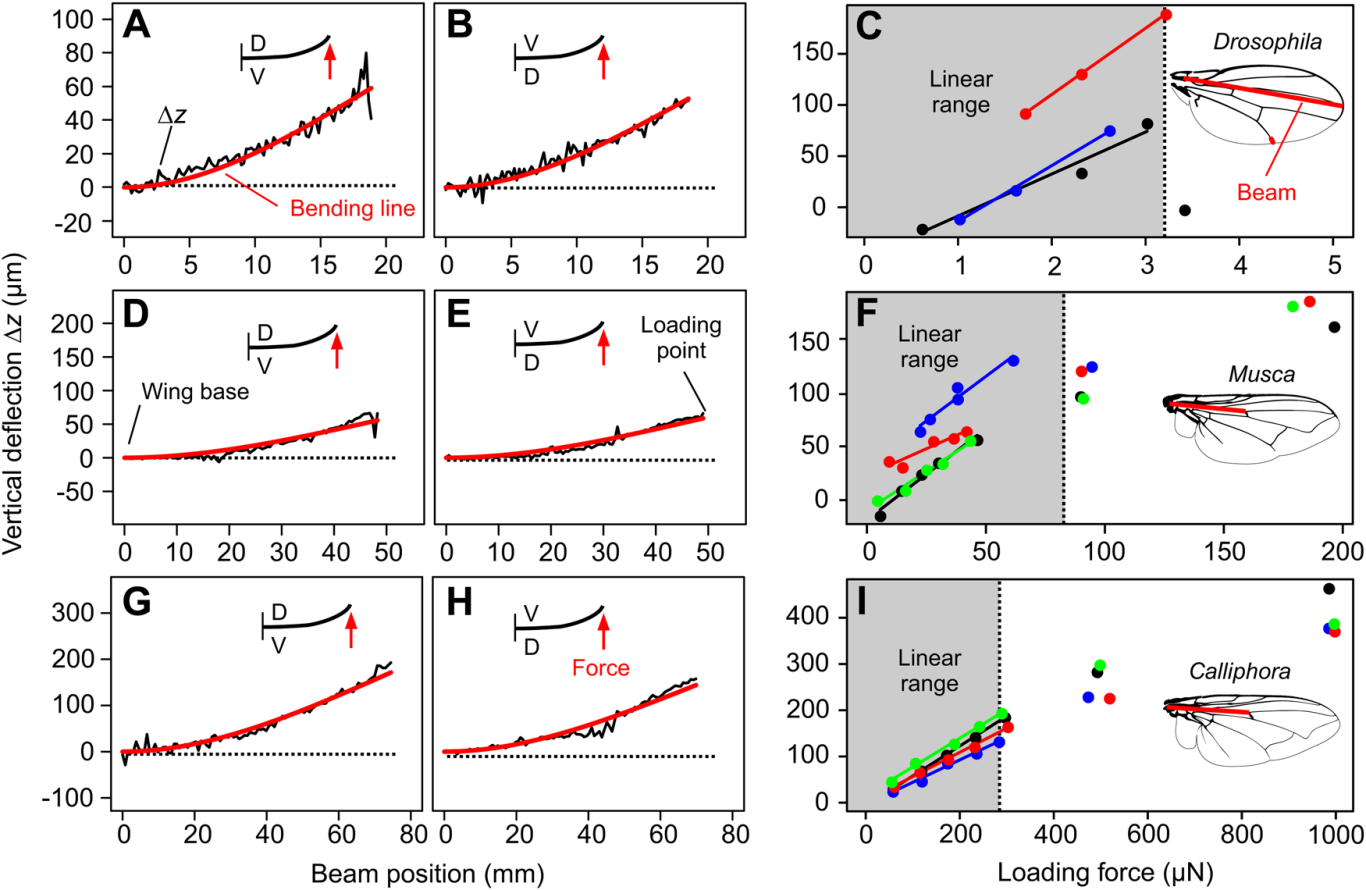
**Determination of spring stiffness using beam theory.** (A,B,D,E,G,H) Difference in vertical wing deflection Δ*z* (loaded-minus-unloaded condition) along a bending line from the wing base to wing tip in *Drosophila* (A–C) and wing base to anterior cross vein in *Musca* (D–F) and *Calliphora* (G–I). Best fit bending line from beam theory is shown in red. Insets show direction of force application. D, dorsal; V, ventral wing side. (C,F,I) Vertical deflection in the dorsal direction during wing loading at the end of the third longitudinal vein (load point 2) in C and at the anterior cross vein (load point 6) in F and I. Dashed line indicates 25% body weight in *Drosophila* and 50% weight in *Musca* and *Calliphora* (cf. Table 1). Colored data are single animals and insets show beam in red.

We estimated wing stiffness by repeatedly applying various forces at the same load point. In *Musca* and *Calliphora* this was the anterior cross vein (point 6, see Materials and Methods). For size reasons, however, it was not feasible to load this vein in *Drosophila* and we thus used the end of the third longitudinal vein instead (point 2, see Materials and Methods, Table 2). Point 6 is close to the center of aerodynamic force in fly wings at 0.56 wing length (Ramamurti and Sandberg, 2007). Left and middle columns of Fig. 4 show that wing deflection, i.e. the difference between loaded and unloaded wings, follows a theoretical, homogenous beam line with an *EI* fitted to the data (red, Eqn 1). For the examples shown in Fig. 4 we determined Pearson's correlation coefficient between the best fit bending line (red) and the data (gray) that ranges from 0.96 to 0.98 (Fig. 4A,B,D,E,G,H). Mean correlation coefficient of all measured data is somewhat lower and amounts to 0.89±0.20 (mean±standard deviation, *N*=285 measurements of 77 flies, three species). Table 3 summarizes force values, spring and flexural stiffness, and correlation coefficients of these data.

**Table 2.**
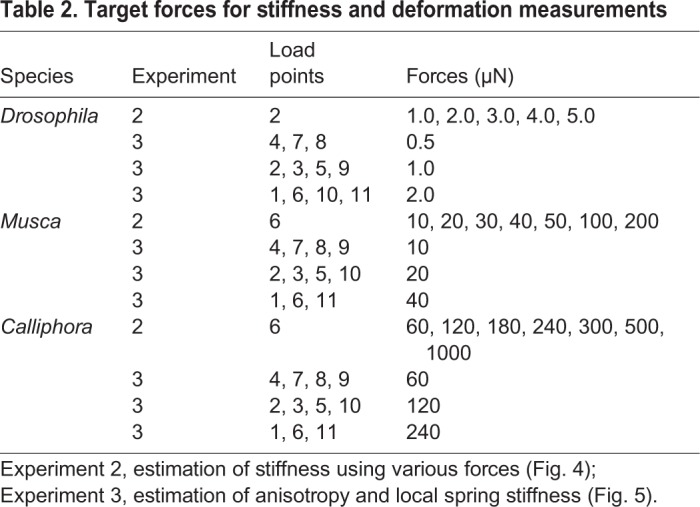
**Target forces for stiffness and deformation measurements**

**Table 3.**
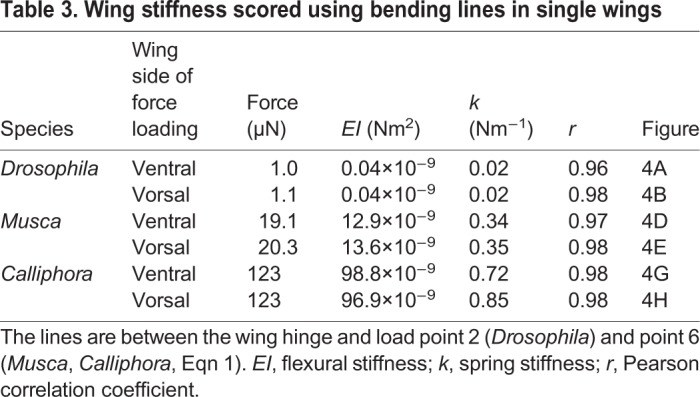
**Wing stiffness scored using bending lines in single wings**

In Fig. 4C,F,I we derived the stiffness from bending at different loads. Within the linear range of values (cut-off value; *Drosophila*, ∼3.3 μN; *Musca*, ∼85 μN; *Calliphora*, ∼350 μN), we estimated stiffness for load point 2 (*Drosophila*) and 6 (*Musca*, *Calliphora*). This analysis yields slopes for *Drosophila* ranging from ∼32.2 m N^−1^ to ∼49.8 m N^−1^, for *Musca* from ∼1.04 m N^−1^ to ∼1.72 m N^−1^, and for *Calliphora* from ∼0.49 m N^−1^ to ∼0.65 m N^−1^ (intercepts, ∼−8 µm–∼30 µm, ∼−20 µm–∼30 µm, ∼−7 µm–∼15 µm; *R*^2^, ∼0.96–∼1.0, ∼0.89–∼0.98, ∼0.99–∼1.0, respectively).

From these slopes we estimated median spring stiffness, i.e. 0.024 Nm^−1^ in *Drosophila* (range, ∼2.00×10^−2^ Nm^−1^ to ∼3.10×10^−2^ Nm^−1^), 0.63 Nm^−1^ in *Musca* (range, ∼58.3×10^−2^ Nm^−1^ to ∼95.8×10^−2^ Nm^−1^), and 1.76 Nm^−1^ in *Calliphora* (range, ∼154×10^−2^ Nm^−1^ to 205×10^−2^ Nm^−1^). For comparison, median flexural stiffness was 4.86×10^−11^ Nm^2^ in *Drosophila* (range, ∼9.2×10^−13^ Nm² to ∼604×10^−13^ Nm²), 9.73×10^−9^ Nm^2^ in *Musca* (range, ∼4.7×10^−9^ Nm² to ∼15.6×10^−9^ Nm²), and 1.33×10^−7^ Nm^2^ in *Calliphora* (range, ∼47×10^−9^ Nm² to ∼500×10^−9^ Nm²). Number of tested wings was 3, 4 and 4, respectively. Notably, we found that spring stiffness calculated from beam theory and regression slopes is not significantly different (two-sided Wilcoxon signed rank test; *P*=0.75, *P*=0.88, *P*=0.88; three species, respectively).

**Fig. 5.**
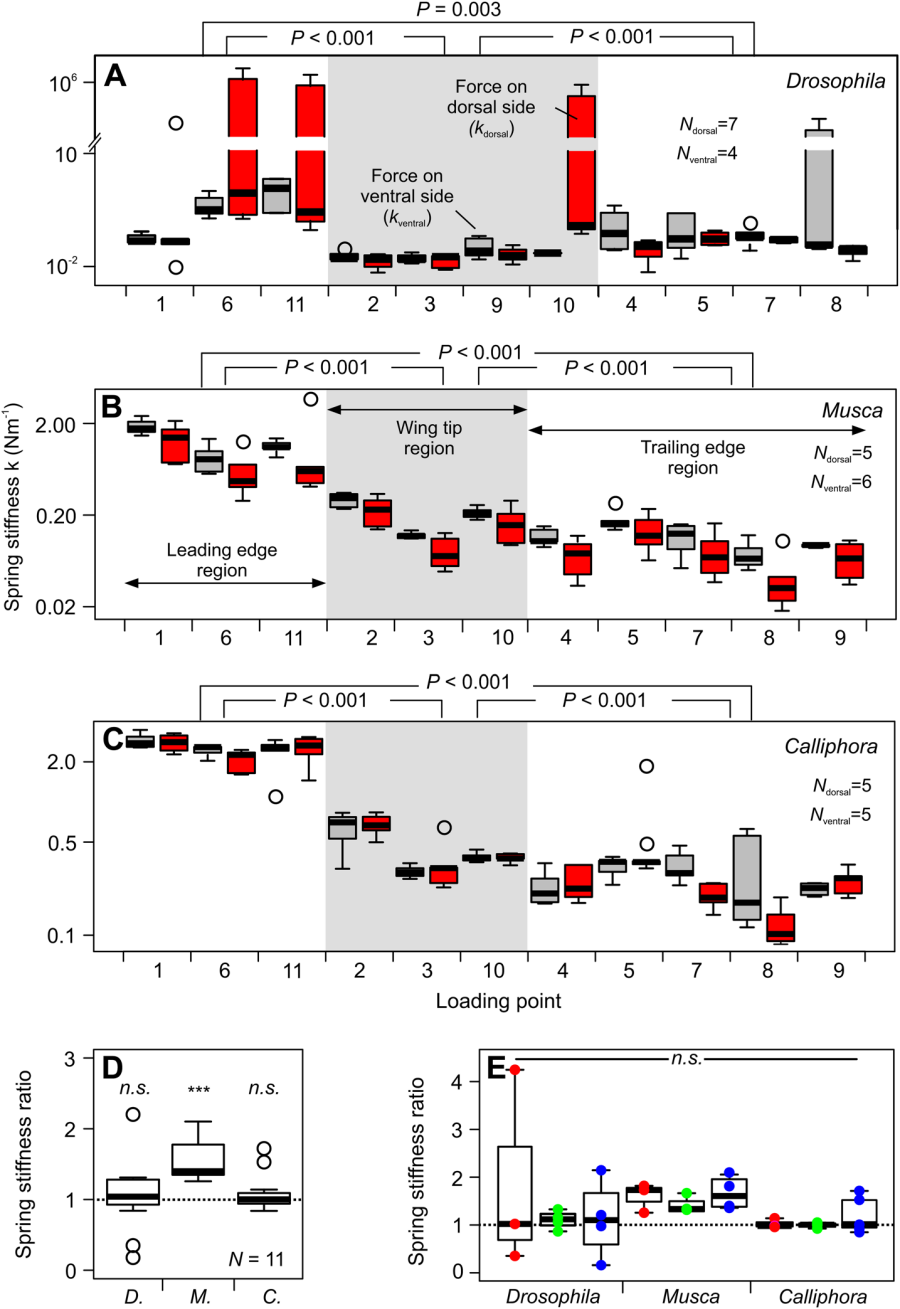
**Wing spring stiffness along various bending lines and anisotropy.** (A–C) Stiffness was calculated along bending lines from the wing base to the various load points. See Table 2 for site-specific target force. Loading forces are applied on the wing’s ventral (gray) and dorsal (red) side. Load points are grouped into three major wing regions (see Materials and Methods). (D) Anisotropy for all loaded points calculated from the ratio in spring stiffness (*k*_ventral_/*k*_dorsal_) derived from loading the ventral (*k*_ventral_) and dorsal wing side (*k*_dorsal_, *N*=11 load points). (E) Anisotropy of three wing regions (red, leading wing edge region, *N*=3 flies, all species); green, wing tip region, *N*=4 *Drosophila*, *N*=3 *Musca*, *N*=3 *Calliphora*; blue, trailing edge region, *N*=4 *Drosophila*, *N*=5 *Musca*, *N*=5 *Calliphora*, see Materials and Methods. All data are boxplots with medians and upper and lower quartiles. Open circles are outliers. ***P<0.001; n.s., not significant. *D*., *Drosophila*; *M*., *Musca*; *C*., *Calliphora*.

Since wing deflection linearly depends on force, we further estimated spring stiffness from single force measurements (Table 2) and along bending lines between wing base and 11 wing positions, respectively (Fig. 5A–C). Taking into account the changing stiffness from the leading to the trailing edge, we applied low forces of 0.5–1.0 µN, 10–20 µN and 60–120 µN for points near the trailing edge, and 2, 40 and 240 µN for points close to the leading wing edges in the three species (Table 2). We found that in all species, wings are significantly stiffer at the proximal leading edge than at both trailing edge (*P*<0.01) and wing tip area (*P*<0.001, Fig. 5A–C). The wing tip area is also stiffer than membranes at the trailing edge (*P*<0.001). Stiffness variance of all bending lines decreases with increasing body size in the tested animals. In *Drosophila*, maximum stiffness is ∼57-fold (force on dorsal side) and ∼77-fold (force on ventral side) larger than minimum stiffness (Fig. 5A). By contrast, in *Musca* these values are ∼44- and ∼26-fold (Fig. 5B) and in *Calliphora* ∼28- and ∼16-fold, respectively.

### Anisotropy

To derive potential anisotropy of wing deflection, we compared the stiffness estimates for deflections in the dorsal (force on ventral side) and ventral (force on dorsal side) direction. Fig. 5D shows that the ratio in spring stiffness of both bending directions is not significantly different from unity in *Drosophila* (median, 1.04; *P*=0.37; *N*=11) and *Calliphora* (median, 1.00; *P*=0.76, *N*=11 test sites). However, in *Musca* the wing is, on average, ∼1.4-times stiffer when bent in the dorsal direction (force on ventral side) than in ventral direction (force on dorsal; median, 1.40; *P*<0.001, *N*=11). This means that in house flies, the wing is more prone to bend during the upstroke than the downstroke, assuming similar forces. We found no significant anisotropy within each of the three wing regions for all species (*P*>0.05, Fig. 5E).

### Estimation of local wing deformation

**Fig. 6.**
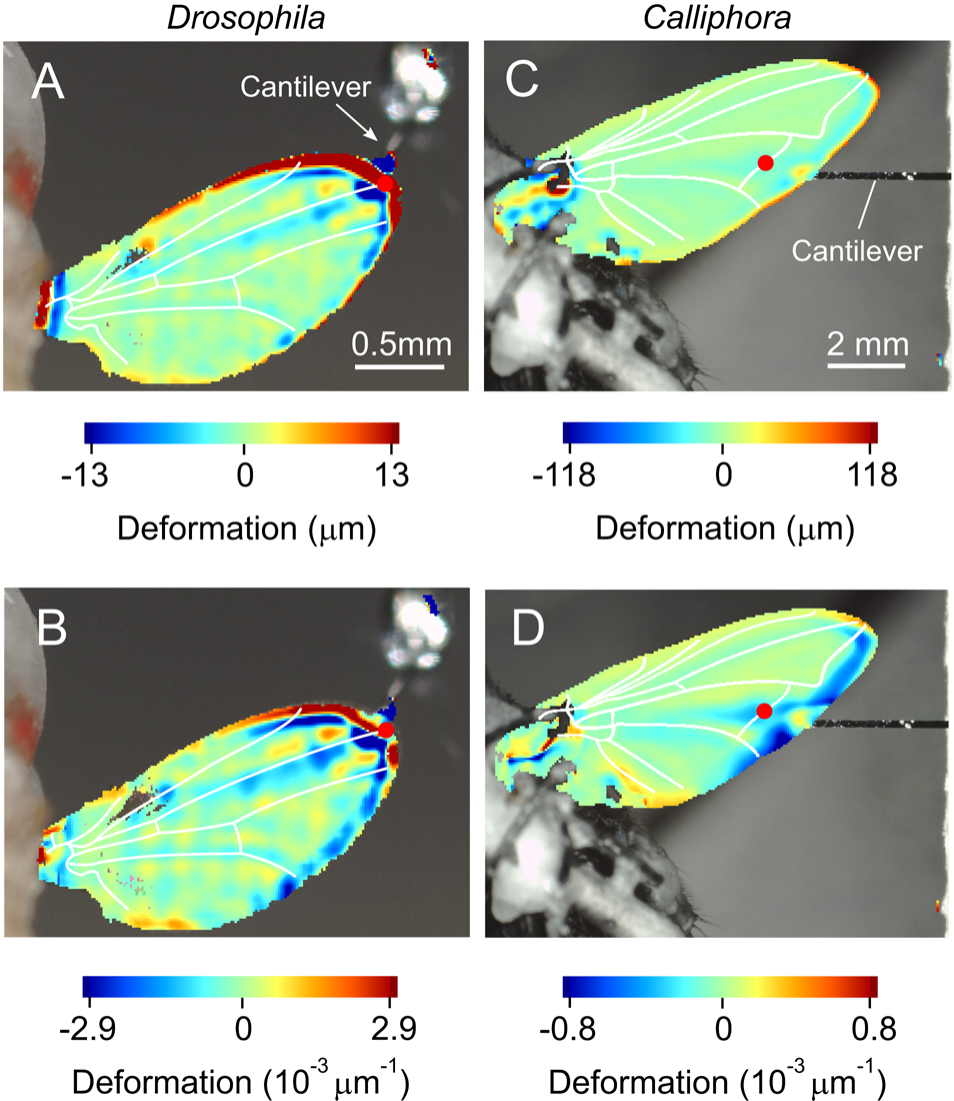
**Local wing deformation derived from two types of analyses (top view on ventral wing surface).** (A,B) Wing is deformed by application of 1.0 μN on the dorsal wing surface at the end of the third longitudinal vein in a single *Drosophila*. (C,D) Images show wing deformation in response to a 138 μN load on the dorsal anterior cross vein in a single *Calliphora*. Deformation was estimated using spatial symmetry approach in A and C and spatial curvature approach in B and D. Forces of 1.0 μN and 138 μN correspond to ∼6% and ∼24% mean body mass, respectively (Table 1). Note the different units of the two methods. Red dots show force load points. Wing veins (white) are superimposed for clarity.

**Fig. 7.**
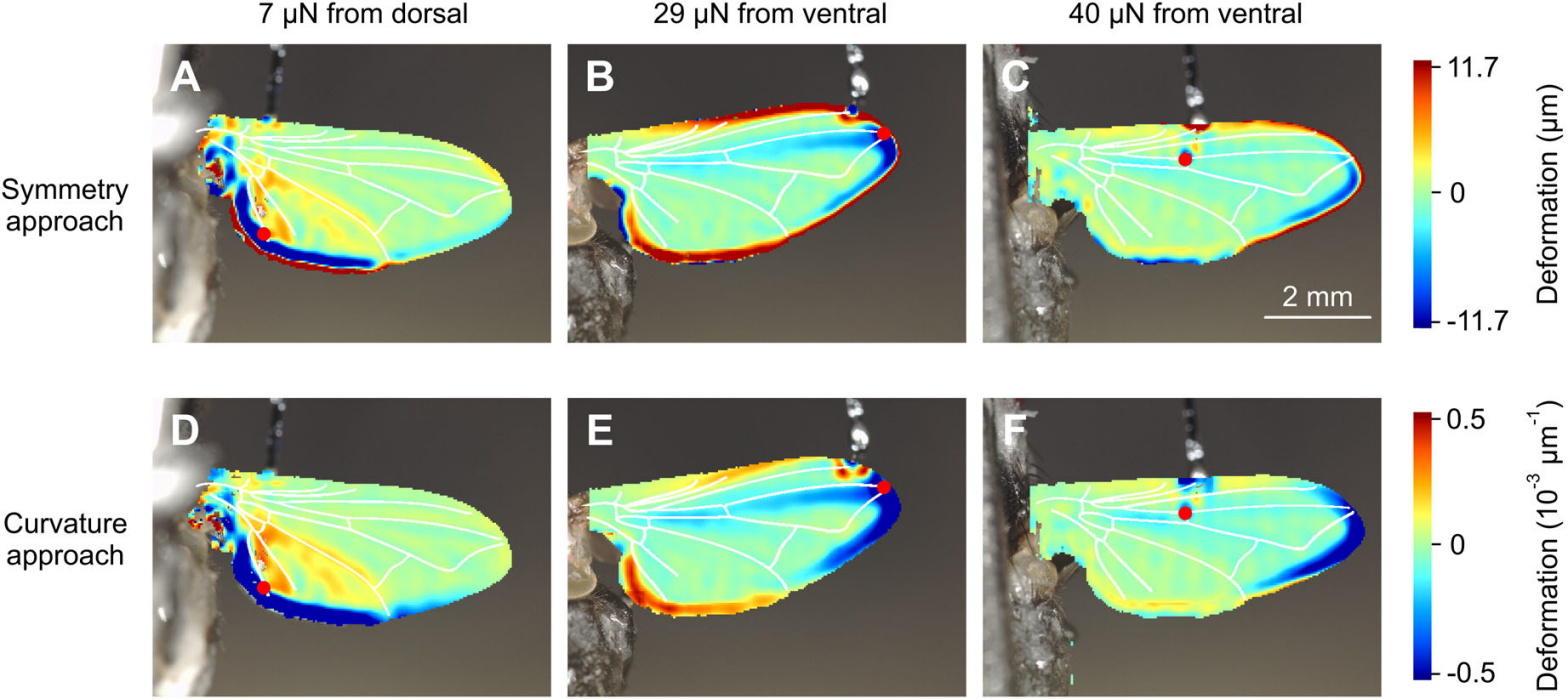
**Local wing deformation at various forces and load points in *Musca*.** Data show local wing deformation in response to (A,D) 7.0 μN load on the dorsal wing side, (B,E) 29 μN on the ventral side and (C,F) 40 μN on the ventral side (top view on ventral side in A,D and dorsal side in B,E,C,F). The latter values correspond to ∼4%, ∼18% and ∼25% mean body weight of this species. Deformation has been calculated using spatial symmetry (upper row) and spatial curvature approach (lower row). Wing veins are superimposed in white. Load points are shown in red.

**Fig. 8.**
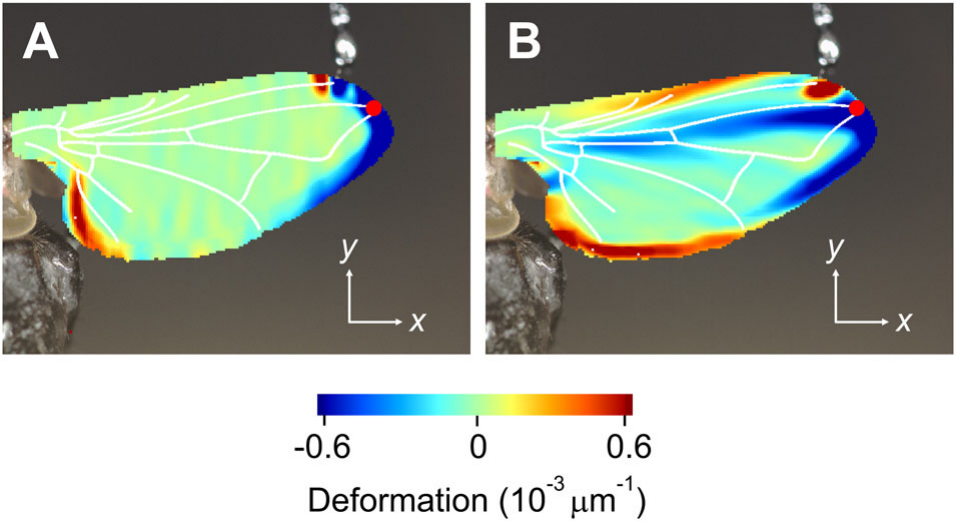
**Wing deformation in *Musca*.** Data show deformation in (A) *x*-direction (along span) and (B) *y*-direction (along chord) of the wing in Fig. 7E. Colors show deformation (spatial curvature approach) at the dorsal wing side. See legend of Fig. 7 for more details.

Figs 6, 7 and 8 show local wing deformation derived from profilometer measurements and using local symmetry and local curvature analyses. All cases show that within the physiological range of flight forces, wing deformation under load is comparatively small and limited to veins and nearby membrane areas. During steady flight, a single wing in *Drosophila* is loaded on average by ∼6–∼8 µN (Table 1). Fig. 6A,B shows that a ∼1.0 µN load at the wing tip leads to deformation of not more than ∼±10 µm. The stress leads to deformation (blue, Fig. 6A,B) of approximately half the third longitudinal vein, but seems to propagate up to the proximal part of the first longitudinal vein, without spreading further. This finding is consistent with the suggested function of the longitudinal vein for bending and torsion control, similar to the findings in *Drosophila*. Fig. 6C suggests that stress from loading the medial (posterior) cross vein in *Calliphora* is transmitted by the fifth longitudinal vein towards the wing base and does not spread further over the entire surface. This load is ∼47% of mean aerodynamic force (∼290 µN) that a single wing needs in order to generate to support body weight. Wing areas at the leading and trailing wing edges are apparently little deformed. We measured a rather uniform deformation along the fifth longitudinal vein of ∼±20 µm.

Fig. 7 shows similar deformation patterns in the house fly *Musca*. A load of 40 µN corresponds to ∼50% the force needed to support body weight by one wing. The load point at the anterior cross vein is close to the expected center of aerodynamic force production (Fig. 7C,F). In Fig. 7B,E, the induced stress apparently buckles the wing at this point and is partly balanced between the proximal parts of the third and fourth longitudinal veins. Fig. 7A,D shows that the deformation in response to forces applied on proximal membranes near the trailing edge is restricted by the fifth longitudinal vein. Membrane areas at the trailing edge may thus deflect in flight without changing the camber of the remaining wing surface. In Fig. 8 we split local deformation of Fig. 7B,E in *x* and *y*-direction. These data suggest that leading edge buckling widely occurs in the chordwise and not in the spanwise direction.

## DISCUSSION AND CONCLUSION

In this study, we determined the mechanical behavior of fly wings in response to static, mechanical loads. The load points were selected according to the wing structure and included veins and membranes. We found that wings that are unloaded but detached from the body rapidly deform with time (Fig. 1). Wings that are attached to the body, by contrast, deform only little within hours and behave similar to homogenous beams (Figs 2–5). The latter finding is quite unexpected because of the wing's complex vein-membrane structure. Notably, we did not find dorso-ventral anisotropy in wings of fruit flies and blowflies, while house fly wings are ∼1.4 times stiffer when pushing against the ventral than the dorsal side (Fig. 5). The latter finding is consistent to previous findings in honeybees suggesting that insect wings are stiffer during the downstroke, when inertial and aerodynamic forces pull at the dorsal wing side during flapping motion (Ma et al., 2017; Ning et al., 2017). A simulation of vein joints in dragonflies suggests that joint spikes and the asymmetric structure of joints are potential sources of anisotropy in the chordwise flexural stiffness of insect wings (Rajabi et al., 2015).

### Significance of experimental procedure

As mentioned in the Introduction, insect wings may undergo tremendous deformation during flapping motion. Since the aerodynamic consequences of wing bending and torsion are under debate (Nguyen et al., 2016), there is an increasing need to understand the wing's mechanical behavior under different load conditions. Previous experimental work applied two approaches to characterize wing deformation: (1) some studies quantified deformation of the entire wing surface during wing flapping motion (Mountcastle and Daniel, 2009; Walker et al., 2009a,b; Koehler et al., 2012). The benefit of this approach is that it catches wing deformation at more natural, dynamic force production. It allows quantification of limits and general characteristics of wing deformation. In most of these studies, however, instantaneous inertial and aerodynamic forces at the wing are unknown. The authors are thus unable to determine local wing stiffness from deformation measurements. (2) Other studies, including the one presented here, tested wings under static loading conditions at which point (Combes and Daniel, 2003a,b) or line (Ganguli et al., 2010; Lehmann et al., 2011) loads are applied to different wing locations or at various distances to the wing hinge, respectively. These measurements allow estimations of wing stiffness but broadly ignore the dynamics of wing deformation behavior. The latter is important because of the visco-elastic properties of resilin at slow deformations (Kovalev et al., 2018). These properties might be the cause for the measured loss of elastic potential energy stored of up to 13% during static wing bending (Fig. 2).

Measurements of force and wing deflection at a single load site typically generate wing stiffness estimates from beam theory, ignoring the complex wing deformation patterns during loading. Combes and Daniel (2003b) tackled this problem by projection of line-patterned lights onto the wing surface that allowed deformation estimation at least of several bending lines. To our knowledge, only one previous study exists that scored wing surface deformation in greater detail during point loading (Yin et al., 2018). The outcome of the latter study, however, is limited because the authors used dried wings (see following section). In our study, we measured deformation in wings attached to living flies and show typical deformation patterns during static loading (Figs 6–8).

### Desiccation and stiffness

There is pronounced variance of stiffness estimates reported for insect wings. Previous authors attributed this variability to desiccation of detached wings because wing stiffness increases up to ∼20-fold with increasing desiccation. This finding has been demonstrated in butterflies (Steppan, 2000; Mengesha et al., 2011), dragonflies (Chen et al., 2013), beetles (Peisker et al., 2013) and flies (Vincent and Wegst, 2004). Fig. 1 quantifies wing deformation with time in the three tested species of flies. Although the detached fly wings were sealed immediately after cutting, they significantly deform within minutes. This is likely due to desiccation and potentially due to the loss of mechanical integrity of the hinge. Although we did not measure the change in stiffness with increasing desiccation, our findings highlight that any stiffness and deformation measurement in insect wings should consider measurements in wings attached to an intact, living insect.

For example: direct estimates of wing stiffness during static and dynamic loading of the hind wing's leading edge in dragonfly (*Sympetrum flaveolum* L.) suggest that Young's modulus changes from ∼30 MPa at static to 615 MPa at dynamic loading (Chen et al., 2013). However, while the latter authors performed static loading measurements in freshly cut wings, the wing was dry during dynamic loading tests. At least part of the reported difference is thus likely due to desiccation. The same holds for experiments using nanoindentation techniques on dried leading edge veins in dragonflies *Pantala flavescens*. The latter study reported stiffness results of 1–2 GPa (Tong et al., 2015). Indeed, a study on the ladybird beetle *Coccinella septempunctata* recently showed that the setal tips contain high amounts of resilin. Since resilin is a hygroscopic protein, it is capable of binding high amount of water (Weis-Fogh, 1960). Nanoindentation experiments on the setal tips thus indicated a Young's modulus of the order of one MPa in the fresh, fully hydrated state, whereas ∼7 GPa after drying (Peisker et al., 2013).

### Stiffness measurements and mechanical properties of the wing

In general, our stiffness estimates are typically smaller than previously reported for isolated fly wings. Data for comparison are available from various sources: (1) the large comparative study on wings of numerous insect species reported spanwise and chordwise flexural stiffness of ∼10×10^−7^ Nm^2^ and ∼50×10^−7^ Nm^2^ for *Calliphora* (Combes and Daniel, 2003a). Other authors found values in detached *Calliphora* wings, amounting to ∼0.01×10^−7^ Nm^2^ at the wing tip and 0.33×10^−7^ Nm^2^ at wing base (Lehmann et al., 2011). Ganguli et al. (2010) reported a spanwise flexural stiffness in *Calliphora* wings of less than ∼0.01×10^−7^ Nm^2^ for the outer second/third distal wing segments including wing tip and up to ∼5.0×10^−7^ Nm^2^ for proximal segments near the wing hinge. These values compare to a median *EI* in *Calliphora vomitoria* (Fig. 5) of 0.35×10^−7^ Nm^2^ between wing root and load point 6. Thus, intact wings might be ∼14-fold (5.0/0.35) more compliant than previously reported for *Calliphora*. Notably, the latter load site at 0.47 wing length distance from the hinge is close to the expected center of aerodynamic force production (0.56 wing length) during wing flapping in flies (Ramamurti and Sandberg, 2007) and may thus be used as a characteristic stiffness estimate for the entire wing. Moreover, the loss of elastic energy during the wing loading-unloading cycle of approximately 10% in all three species (Fig. 3) is approximately half of the loss in detached wings of *Calliphora* (20–23%, Lehmann et al., 2011). Assuming that this loss is due to creep deformation, a living wing is not only more compliant but also significantly more elastic than a desiccated wing.

Our study did not primarily intend to explain how forces are distributed by veins and membranes in insect wings. Figs 6–8 suggest that local deformation patterns can be quite complex and greatly depend on the location at which loads are applied. The anal lobe, for example, can deform rather independently of the rest of the wing and veins can act as hinges. Surface buckling along the third longitudinal vein, for example, might help to control wing camber (Figs 7E and 8B). Nevertheless, a comprehensive description of local deformation requires 3-dimensional deformation data, while the profilometer only recorded changes in the vertical *z*-direction. Owing to changes in optical reflection of the wing surface during loading, our mathematical algorithms were not able to reliably generate these data. Similar problems have previously been reported in a deformation study on insect wings (Yin et al., 2018).

### Stiffness scaling

We found that spring (*k*) and flexural stiffness (*EI*) increases with increasing body weight, which is in accordance to a previously published large comparative study (Combes and Daniel, 2003a). We found that at loads near the center of aerodynamic force production (point 6, see Materials and Methods), wings of large flies (*Calliphora*) are ∼8.1 times (force on ventral side) and ∼2.5 times (force on dorsal side) stiffer (spring stiffness) than in small flies (*Drosophila*). Mean wing stiffness ratio between both species is 15.6±18.2 (mean±standard deviation, *N*=11 load sites). This difference is expected owing to the larger body mass in *Calliphora*. However, the ratio between median spring stiffness (force on ventral wing side, Fig. 4C,F,I) and body mass (Table 1) does not increase likewise. These ratios are 0.015 (0.02 Nm^−1^, 1.6 mg) in *Drosophila*, 0.038 (0.63 Nm^−1^, 16.4 mg) in *Musca* and 0.030 (1.76 Nm^−1^, 59.3 mg) in *Calliphora*. We conclude that wing stiffness in flies does not isometrically scale with changing body size but might depend on the changing requirements for flight in flies instead. By contrast, our data suggest that variance in spring stiffness of the 11 bending lines decreases with increasing body mass from ∼77-fold in small flies (*Drosophila*) to ∼55-fold (*Musca*) and ∼28-fold (*Calliphora*) in large flies. Spring stiffness homogeneity is thus approximately three times larger in large fly wings than in small fly wings. To our knowledge, the consequences of these differences for aerodynamic force production are yet unknown.

Altogether, the results of this study highlight the complexity of insect wing design for span- and chordwise stiffness. Owing to the static loading approach, our estimates should be seen with caution and may not be directly applied to explain wing deformation occurring during wing flapping. Nevertheless, the data are helpful for understanding elastic material properties of insect wings that in turn are of importance for validation of physical and numerical wing models. These models are currently under construction by engineers and mathematicians. We also believe that our data provide a solid basis towards the further design of biomimetic flight vehicles (Shyy et al., 1999, 2008). It has previously been suggested that flight of these vehicles is limited by power and the ability to produce elevated lift, but also by the robustness of their wings during motion at high frequencies (Stafford, 2007; Bontemps et al., 2012; Ma et al., 2013).

## MATERIALS AND METHODS

### Animals and preparation

All data were collected from a total of 34 female fruit flies *Drosophila melanogaster* Meigen, 35 female house flies *Musca domestica* Linnaeus and 34 female blowflies *Calliphora vomitoria* Linnaeus*.* The number of tested flies varied among the three main experiments as shown in Table 1. The animals were cold-anaesthetized, attached to a hypodermic needle and eventually fully coated using eicosane (CAS: 112-95-8, Sigma-Aldrich) to delay desiccation. In experiments on shape changes of attached and detached wings, we used clear nail varnish (essence cosmetics, cosnova GmbH, Sulzbach, Germany) instead of eicosane. The right or left wing was extended and fixed at the base for stability. The animals were tested immediately after preparation and remained alive throughout the experiments. Eicosane melts at ∼37°C; well below maximum flight muscle temperature during flight in *Calliphora* (∼42°C, Stavenga et al., 1993). It is commonly used in insect research (Vallet et al., 1992; Szyszka et al., 2005; Haehnel et al., 2009; Yamagata et al., 2009; Chakroborty et al., 2016).

### Experimental setup and procedures

**Fig. 9.**
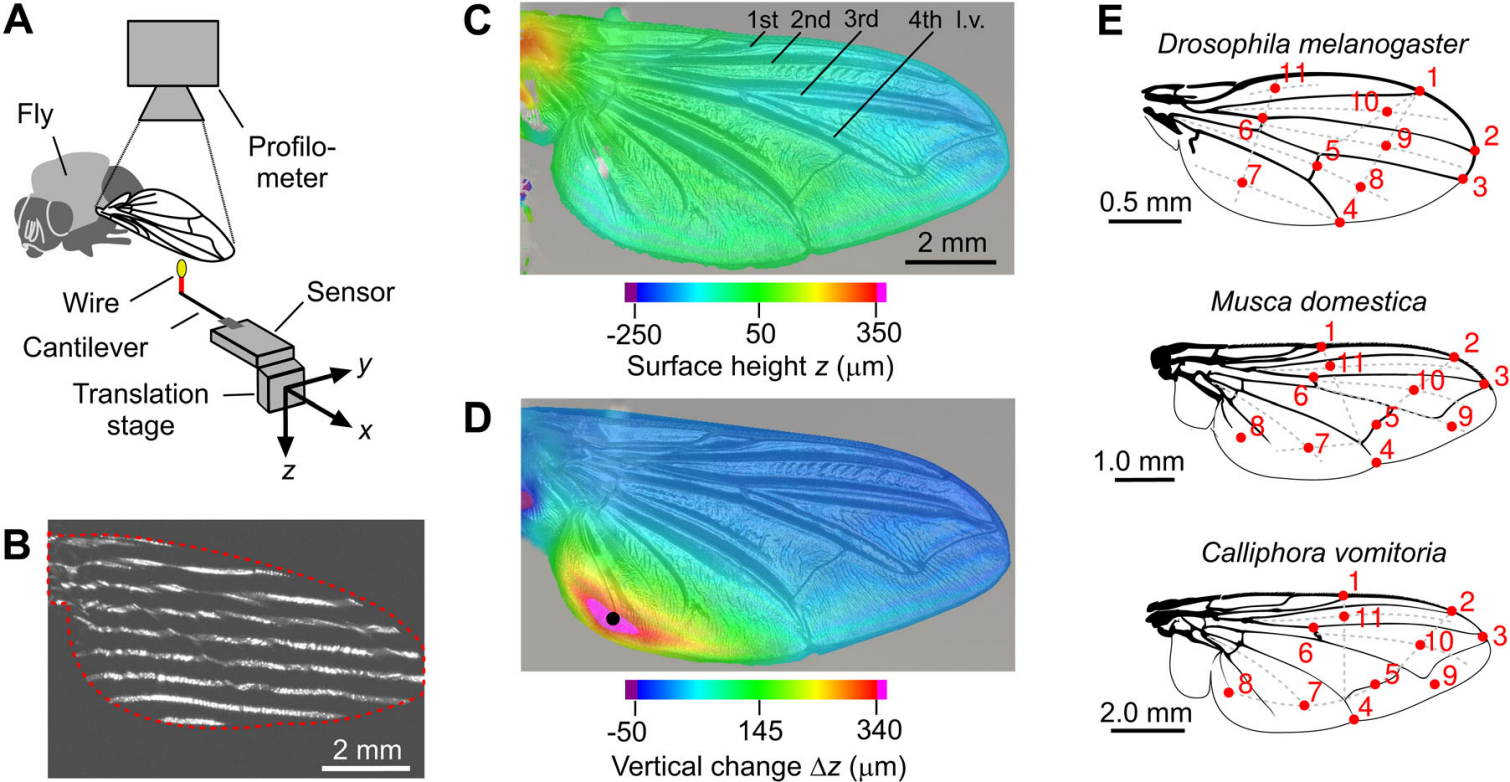
**Experimental setup and load points.** (A) Fly wings of intact animals are positioned below an optical profilometer. A force sensor is mounted to micro-translation stage. A wire (red) is attached to the sensor's cantilever and painted with fluorescent dye (yellow). (B) Example of a video image showing a visual line pattern of the profilometer on the wing during surface scanning (wing shape, red). (C) Surface scan result of an unloaded wing and (D) the changes in vertical deflection (Δ*z*) in response to a 7.0 μN point load at point 8 (black dot, *Musca*, top view on ventral side). Positive (red, higher) and negative (blue, lower) *z*-values indicate vertical positions with respect to a horizontal mean. (E) Load sites during force application. Dots indicate load sites on both dorsal and ventral wing surfaces. When loading the dorsal surface, the ventral side of the wing was up and vice versa. Location of points at membrane areas are supported by imaginary auxiliary lines (dotted). l.v., longitudinal vein.

We mounted the tethered fly to a holder below an optical profilometer (VR-3000, Keyence Corporation, Osaka, Japan) that recorded local vertical height of the entire wing surface (Fig. 9A–D). Point forces were applied and measured by a small, cantilever-based force sensor with nanonewton precision (CiS Forschungsinstitut für Mikrosensorik GmbH, Erfurt, Germany). The sensor was attached to an *xyz*-micro-translation stage (M-111.12S micro-translation stages, Physik Instrumente, Karlsruhe, Germany) and positioned by software (PIMikroMove). We glued a tungsten wire with a diameter of 50 µm to the sensor's cantilever, painted the wire tip with yellow fluorescent dye to enhance visibility, and used the wire tip to apply point forces. We calibrated the force sensor prior to the experiment by attaching known weights (wire loops) of 0.8 mg, 1.3 mg, 2.09 mg, 3.1 mg and 5.18 mg to the cantilever. Mean ambient temperature during the experiments was 26°C and ambient relative humidity 29% (Table 1).

After aligning the animal, we positioned the force transducer near one of up to six veins (first to fifth longitudinal veins, anterior and posterior cross veins) and five membrane positions (marginal, submarginal and posterior cells) just below the wing surface (Fig. 9E) and started a modified version of a custom-made software routine written in LabVIEW (National Instruments; software provided by CiS Forschungsinstitut). We grouped the load points into three regions (Fig. 9E): (1) a *leading edge region* that includes load points 1, 6, 11 (all tested species); (2) a *wing tip region* with points 2, 3, 9, 10 (*Drosophila*) and 2, 3, 10 (*Musca, Calliphora*); and (3) a *trailing edge region* with points 4, 5, 7, 8 (*Drosophila*) and 4, 5, 7, 8, 9 (*Musca, Calliphora*).

The software moved the sensor wire against the wing surface at constant velocity of ∼2 µm s^−1^. Stage movement automatically stopped when the sensor reached the desired load. The applied target forces were set according to structural and aerodynamic considerations, and the animals' body masses (Tables 1 and 2). In experiments, in which we applied different forces to the same wing location, force sequence was increasing or decreasing but with the two largest forces always tested last. This was done to mitigate any influence of sample ageing while avoiding wing damage owing to elevated forces. In experiments, in which we applied a single force to different locations, we randomized the order of testing sites. Force data were recorded using a USB-6009 data acquisition device (National Instruments) at mean sample rate of 2.5 samples per second.

**Fig. 10.**
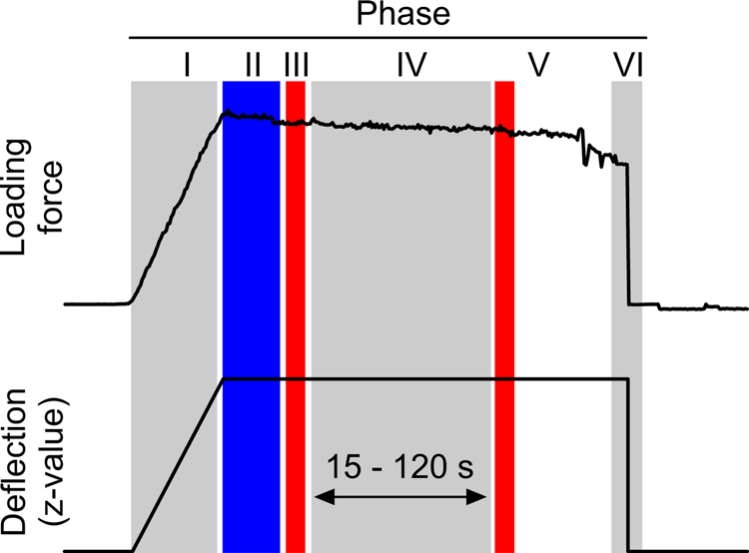
**Force-time and deflection-time curves obtained from force measurements and motor motion, respectively.** The sensor exerts a load while moving against the wing surface (phase I). During phase II, the sensor does not move and at phase III five force values are measured. At phase IV, the profilometer light is switched on and the wing surface is scanned. Profilometer readings stop and post-measurement forces are recorded at phase V. Sensor moves back and releases the wing surface at phase VI. Typical measurement time for the entire procedure was 90–120 s.

We scored forces at the earliest 10 s after the sensor stopped moving and the force signal became stable. Owing to heat-induced drift of the force sensor by the profilometer light, we could not reliably determine forces during the deformation measurements and thus averaged the value from up to five consecutive measurement values before and after (red, Fig. 10) the profilometer illumination. In cases in which both measures were off by more than 20%, we dismissed the data. For control, profilometer measurements on unloaded wings were conducted between each wing loading experiment. A full optical recording was 15–120 s and total measurement time for a single wing ranged from 45–60 min. All data were processed using the statistical programming language R (Version 3.3.3) including various extensions (R Foundation for Statistical Computing, www.r-project.org, see Supplementary methods). If not stated otherwise, data are medians and statistical tests are conducted on medians using two-sided Wilcoxon-Mann–Whitney test.

### Estimation of stiffness

We determined spring stiffness of the tested wings using two approaches: (1) bending lines and (2) regression analysis between loading force and wing deflection. We use bending lines in cases in which we only applied a single load at the wing. Stiffness estimates that rely on a single force/deflection ratio are more prone to measurement errors but allowed us to show that the wing broadly behaves like a simple homogenous beam. A higher accuracy of stiffness estimates we obtain from the relationship between loading force and wing deflection in cases, in which the same wing position was loaded with different forces. In the latter case, spring stiffness was derived from the slope of standard major axis regression analysis (model 2 regression) to surface deflection (*y*-value) and loading force (*x*-value).

Stiffness from bending lines was conducted using beam theory. This procedure was as follows: we first considered the beam along a straight line in the *xy*-plane from the humeral cross vein (hinge) to the load point (Fig. 9E). We divided the bending line into 100 points (*x*-positions) and calculated wing deflection from the difference in surface height (Δ*z*) between loaded and unloaded conditions. The bending line of a cantilevered beam is:
(1)
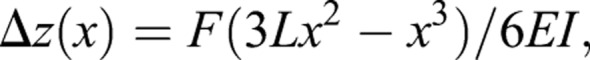


with *F* the applied force at the beam end, *L* the total length of the bending line (beam length), *x* the position along the beam and *EI* the flexural stiffness (Oberg et al., 2000). We used this equation to fit the bending line to the data, minimizing least mean square error (LMSE). Spring stiffness (*k*) was eventually derived from flexural stiffness using the equation (Oberg et al., 2000):
(2)
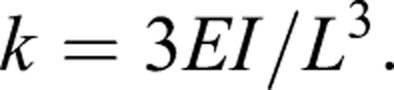


### Calculation of local wing deformation

**Fig. 11.**
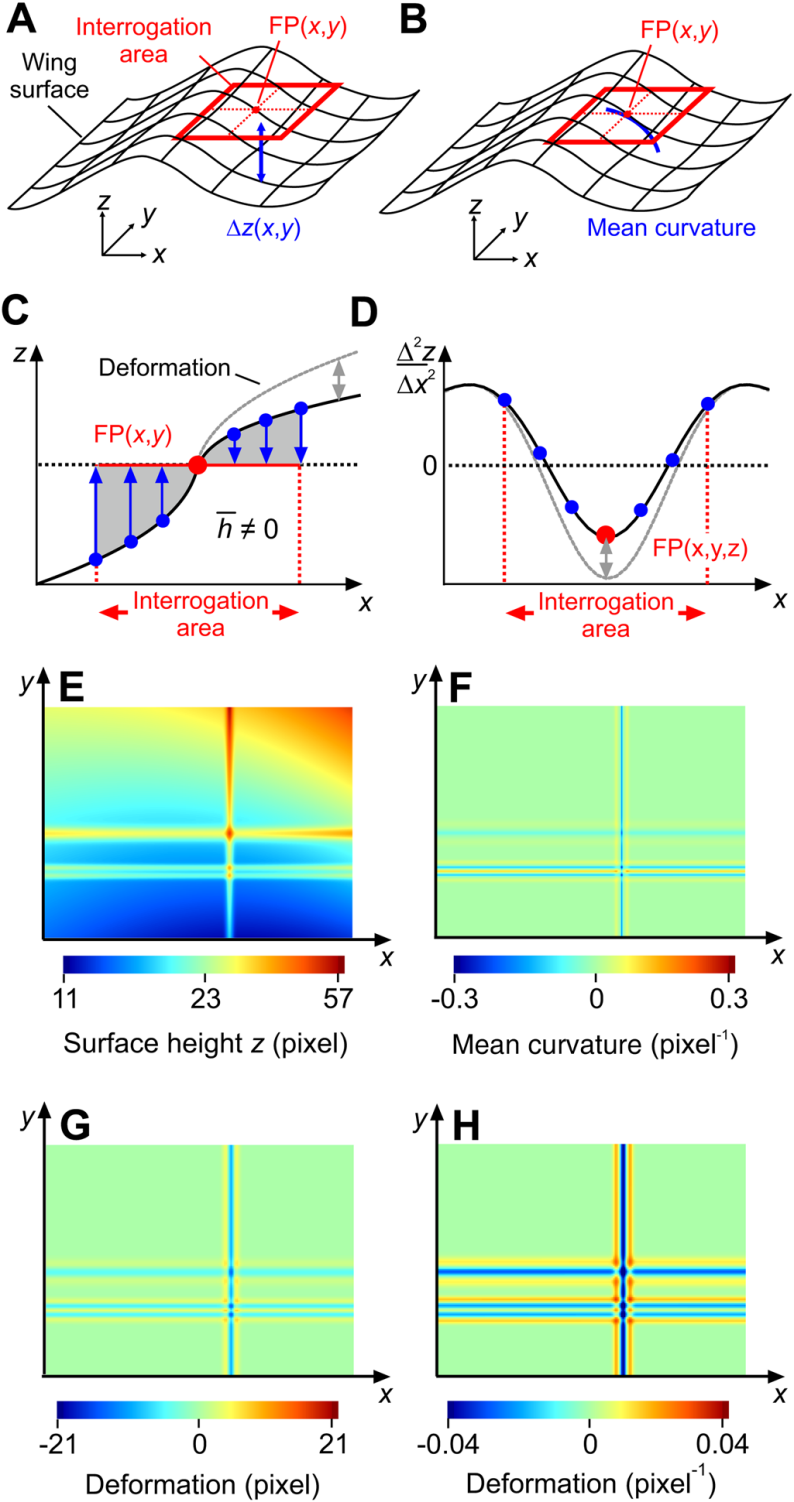
**Deformation analyses of wing surface.** 3-dimensional schematics of (A,C) spatial symmetry and (B,D) spatial curvature approach. Image size is 768×1024 pixels. *z*, vertical deflection; FP, focal point. See text for more information. Deformation (gray) is shown in 2 dimensions in C and D for clarity. (E) Color-coded surface height (*z*-values) produced by the test function that is specified in the Supplementary methods (Table S1). (F) Deformation is calculated as mean surface curvature from derivatives (Eqn 7) of the test function (not the data). Deformation estimates using the test data in E (not the function) are shown in (G) based on the spatial symmetry approach and in (H) the spatial curvature approach.

Local wing deformation is defined as the local change in wing shape between loaded and unloaded conditions. We here introduce two approaches to determine local wing deformation from profilometer measurements: (1) an approach based on the local symmetry of the wing surface (spatial symmetry approach, Fig. 11A,C) and (2) an approach based on the estimation of mean surface curvature (spatial curvature approach, Fig. 11B,D). Both approaches produce similar deformation data and are similarly constrained, e.g. at data near the wing edges. A numerical test gives credence to the validity of both procedures (Fig. 11E–H, see below).

Due to changing reflections on the loaded and unloaded wing surface, the optical profilometry did not provide consistent displacement fields between measurements. However, since the maximum *xy*-displacement of corresponding wing structures was not more than 15 image pixels or ∼2% wing length between images, we ignored image distortions and smoothed the images, applying seven times a disk filter with a radius of 10 image pixels in *x*- and *y*-direction before calculation of deformation. This blurring procedure partly eliminated the significance of local image distortions by a reduction of spatial image resolution. In both numerical approaches used for the calculation of local deformation, the size of the interrogation area was 61×61 pixels (see Supplementary methods, Fig. S1). Considering a pixel size of ∼2.47 µm, ∼7.40 µm and ∼11.8 µm in images of the three fly species, the latter values convert into ∼2.27×10^−2^ mm^2^ in *Drosophila*, ∼20.4×10^−2^ mm^2^ in *Musca* and ∼81.8×10^−2^ mm^2^ wing surface in *Calliphora*. See Supplementary methods for more details.

### Spatial symmetry approach

The local symmetry approach is based on the estimation of mean surface height that is calculated from the sum of all *z*-values relative to the focal point FP within the interrogation area (Fig. 11A). The model is broadly independent of the surface shape and may be applied to both flat and curved surfaces. Surface translation and small rotations about FP produce only negligible changes in mean height. For each interrogation area that slides over the wing surface with a step width of five image pixels in *x*- and *y*-direction, we calculated mean height (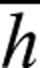) from equation:
(3)
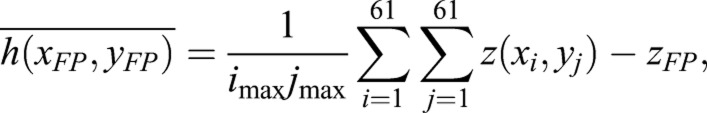


with *z_FP_* the *z*-value of the focal point, and *x_i_* and *y_j_* the orthogonal coordinates running from pixel 1 to 61 inside each interrogation area. The coordinates *x* and *y* are in the horizontal and the wing surface was approximately parallel to *x*-and *y*-axes. If the wing deforms under load, the spatial balance in *z*-values changes about FP. Local wing deformation (*D_H_*) based on mean height is thus:
(4)
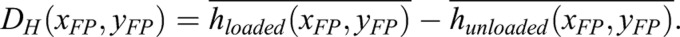


Negative deformation values indicate that the wing is locally bent and cambered downwards. Wing deformation based on symmetry has the unit ‘length’.

### Spatial curvature approach

Mean curvature of the wing surface (*C_m_*) is defined as:
(5)
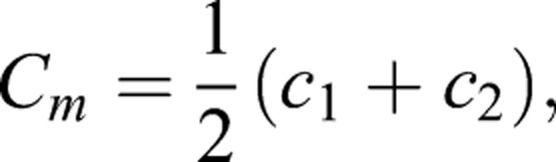


with *c*_1_ and *c*_2_ the principal curvatures orthogonal to each other (Fig. 11B). We estimated curvature from a 2-dimensional function fitted to the *z*-values in the interrogation area about FP. We used the following second order linear model to the surface *z*-values for approximation of local curvature, i.e.:
(6)
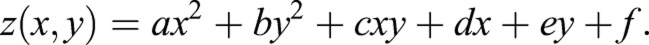


We determined the coefficients *a*–*f* in this equation using all *z*-values inside the interrogation area and solving the appropriate matrix using a least mean square error function (lm-function in R). The function allows to calculate local *z* for each *xy*-point of the wing surface. The first and second derivatives of the fit function in Eqn 6 yield mean curvature *C_m_*, i.e.:
(7)
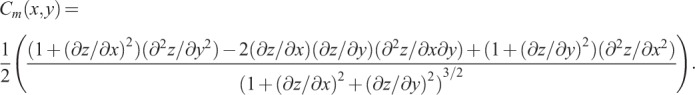


Similar to the symmetry approach, local wing deformation *D_Cm_* based on curvature eventually equals:
(8)
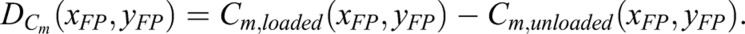


Step size for the moving interrogation area was five image pixels and similar to the local symmetry approach. Positive curvature indicates a bowl-like wing shape and negative curvature an inverted bowl-like shape. Since curvature equals 1/radius, wing deformation based on curvature has the unit ‘1/length’.

### Validation of methods

We validated the two types of wing shape calculation by generating surface data at the scale of typically structured fly wing veins, using a numerical test function (see Supplementary methods, Table S1). The test function's *z*-values are shown in Fig. 11E. The distance between the colored lines in Fig. 11E are broadly similar to characteristic distances between wing veins in *Musca*. Fig. 11F shows mean curvature of the test function that was calculated from the derivatives in Eqn 7. For comparison, mean height values of the spatial symmetry approaches are calculated from the data of the test function and shown in Fig. 11G. Fig. 11H shows mean curvature of the spatial curvature approach and using Eqn 7. Again, here we used the data of the test function to determine the parameters of Eqn 6. We found that, to a large extent, the numerical solution matches the results of the two approaches. More details on the methods are presented in the Supplementary methods.

## Supplementary Material

Supplementary information
