# Supplementary material for: Local deformation and stiffness distribution in fly wings
Source: Biol Open. 2019 Jan 15;8(1):bio038299. doi: 10.1242/bio.038299 (PMC6361194; doi:10.1242/bio.038299)
Supplement: Supplementary information [file biolopen-8-038299-s1.pdf]

Supplementary methods

Statistical comparison

The procedure below statistically compares the two approaches we used for calculation of local wing deformation, i.e. spatial symmetry approach and spatial curvature approach. The procedure also evaluates the significance of the interrogation window (IW) size and data filtering.

The evaluation was as follows: (1) We first generated specific *z*-values using three test functions for an 1024 pixel x 768 pixel image (see below, eq. S1, image 1). (2) From each *z*-value image, we derived a second image (image 2) by shifting the first image by 11 pixels in *x*- and *y*-direction. Both images were then cut to their overlapping area, resulting in 1013 pixel x 757 pixel image. Copies of both images were smoothed applying 7-times a disk filter with a radius of 10 image pixels, similar to the procedure mentioned in the manuscript. Deformation was calculated using the symmetry approach (app 1) and curvature approach (app 2) for all unfiltered and filtered *z*-value images, which yields a total of 4 images with deformation data.

In a next step, we plotted all *z*(x,y)-values of image 1 (unfiltered data, app 1) against image 2 (unfiltered data, app 1), and calculated the mean Pearson correlation coefficient. The latter procedure was repeated using the second curvature approach (app 2) and also data of each fly species. Figure S1 (left) shows the coefficients as a function of interrogation window size. The data suggest that both approaches yield poor coefficients for IW sizes below ~40 pixel x 40 pixel. At higher IW sizes, the coefficients saturate below ~0.8. At IW sizes above ~70 pixel x 70 pixel, the spatial curvature approach produces higher coefficients than the symmetry approach.

Similar to the procedure above, we also calculated the mean Pearson correlation coefficient for the filtered data. Figure S1 (right) shows that with filtered data, the symmetry approach yields higher coefficients than the curvature approach for small IW sizes ranging from 15 pixel x 15 pixel to 60 pixel x 60 pixel. Both methods converge at IW sizes above 60 pixel x 60 pixel at elevated coefficients of ~0.7 – ~0.8. In general, coefficients derived from filtered data were significantly higher than the coefficients for the unfiltered data. Based on these findings, we decided to filter our measured wing surface data (*z*-values) and selected an IW size of 61 pixel x 61 pixel for our deformation analysis.

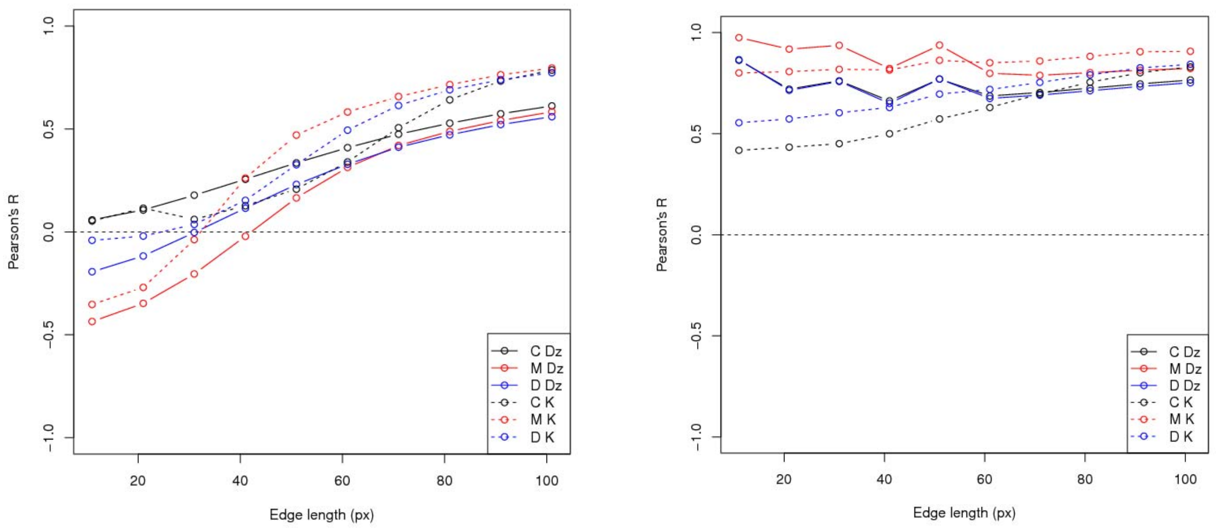

Fig. S1. Pearson correlation coefficient plotted against edge length of the interrogation window. (Left) Coefficients for unfiltered data. (Right) Coefficients for filtered data. C, *Calliphora*; M, *Musca*; D, *Drosophila*; Dz, spatial symmetry approach; K, spatial curvature approach.

Biology Open • Supplementary information

Test function

For testing our deformation analyses, we developed a numerical function that generated 768 x 1024 wing surface values (*z*-values, 786432 image pixels). The function mimics *z*-values that are typical for the vein-membrane structure of wings in *Drosophila*, *Musca,* and *Calliphora*. Color-coded *z*-values computed for *Musca* are shown in figure 3E of the manuscript. The test function was:

$$z(x,y) = a_1x + a_2y + a_3xy - b_1 sin(b_2x) + f_1c_1 + f_1c_2 + f_2c_3 + f_2c_4 , \quad \text{(eq. S1)}$$

with

$$c_1 = exp\left(-\frac{(y-m_1)^2}{2g_1^2}\right),\ c_2 = exp\left(-\frac{(y-m_2)^2}{2g_1^2}\right),\ c_3 = exp\left(-\frac{(y-m_3)^2}{2g_2^2}\right),\ c_4 = exp\left(-\frac{(x-m_4)^2}{2g_1^2}\right).$$

The parameters *x* and *y* are the *xy*-position of an image pixel. Parameter values are shown in table S1.

Table S1. Parameter values of the test function. x_{max}, image size in *x*-direction (1024), y_{max}, image size in *y*-direction (768).

Parameter	Significance	*Drosophila*	*Musca*	*Calliphora*
a_1	wing orientation	$30\,x_{max}^{-1}$	$30\,x_{max}^{-1}$	$50\,x_{max}^{-1}$
a_2	wing orientation	$30\,y_{max}^{-1}$	$30\,y_{max}^{-1}$	$50\,x_{max}^{-1}$
a_3	wing orientation	$30\,x_{max}^{-1}\,y_{max}^{-1}$	$30\,x_{max}^{-1}\,y_{max}^{-1}$	$50\,x_{max}^{-1}\,y_{max}^{-1}$
b_1	wing curvature	35	35	50
b_2	wing curvature	$\frac{\pi}{2048}$	$\frac{\pi}{2048}$	$\frac{\pi}{2048}$
f_1	vein height	8	15	30
f_2	vein height	60	20	110
g_1	vein half width	8	8	10
g_2	vein half width	12	15	12
m_1	vein position *y*	200	200	200
m_2	vein position *y*	230	225	220
m_3	vein position *y*	320	335	330
m_4	vein position *x*	600	600	600

Software extensions

For the statistical analyses, we used the following "R-packages": *abind* (Plate and Heidberger, Version 1.4-5, 2016), *akima* (Akima and Gebhardt, Version 0.6-2, 2016), *jpeg* (Urbanek, Version 0.1-8, 2014), *oro.dicom* (Whitcher et al., Version 0.5.0, 2011), *colorRamps* (Keitt, Version 2.3, 2012), *smoothie* (Gilleland, 1.0-1, 2013), and *tiff* (Urbanek, Version 0.1-5, 2013).

Biology Open • Supplementary information
